# Good News or Bad News, Which Do You Want First? The Importance of the Sequence and Organization of Information for Financial Decision-Making: A Neuro-Electrical Imaging Study

**DOI:** 10.3389/fnhum.2018.00294

**Published:** 2018-07-27

**Authors:** Wenting Yang, Jianhong Ma, Hezhi Chen, Anton G. Maglione, Enrica Modica, Dario Rossi, Giulia Cartocci, Marino Bonaiuto, Fabio Babiloni

**Affiliations:** ^1^Department of Psychology and Behavioral Science, Zhejiang University, Hangzhou, China; ^2^Department of Molecular Medicine, Sapienza University of Rome, Rome, Italy; ^3^Department of Psychology of Development and Socialization Processes, Sapienza University of Rome, Rome, Italy; ^4^Department of Computer Science, Hangzhou Dianzi University, Hangzhou, China

**Keywords:** hedonic editing hypothesis, order effects, financial decision-making, EEG, approach/avoidance, emotion, LORETA

## Abstract

Investment decisions are largely based on the information investors received from the target firm. Thaler introduced the hedonic editing framework, in which suggests that integration/segregation of information influence individual's perceived value. Meanwhile, when evaluating the evidence and information in a sequence, order effect and biases have been found to have an impact in various areas. In this research, the influence of the Organization of Information (Integration vs. Segregation) and the Sequence of Information (Negative-Positive order vs. Positive-Negative order) on individual's investment decision-making both at the behavioral level (decision) and neurometrix level (measured by an individual's emotion and Approach Withdraw tendency) was assessed for the three groups of information: a piece of Big Positive Information and a piece of Small Negative Information, a piece of Big Negative Information and a piece of Small Positive Information, and a piece of Small Negative information. The behavioral results, which are an individual's final investment decision, were consistent for all three scenarios. In general, individuals will invest more/retire less when receiving two pieces of information in a Negative-Positive order. However, the neurometric results (Emotional Index, Approach Withdraw Index and results from LORETA) show differences among information groups. An effect of the Sequence of Information and the Organization of Information was found for the different scenarios. The results suggest that in the scenarios that involve large-scale information, the organization of information (Integration vs. Segregation) influences the emotion and Approach Withdraw tendency. The results of this investigation should provide insight for effective communication of information, especially when large-scale information is involved.

## Introduction

Investment decisions are largely based on the information investors receive from target firms (Clor-Proell, 2009). These pieces of information predict actual and potential gains and losses. For individual gains and losses, Thaler (1985, 2018) introduced the notion of hedonic editing. The framework of hedonic editing was derived from Kahneman and Tversky (1979) prospect theory, based on the assumption that in performing mental accounting, individuals tend to frame multiple forms of information in a manner that results in the highest perceived value. The hedonic framing hypothesis suggests that, in some situations the highest perceived value may be achieved by integration of information, whereas in others situations the segregation of multiple forms of information can maximize perceived value. More specifically, the form of the information is better accepted if; (1) a decrease in gain was presented in an integrated manner, (2) a small reduction in loss was presented in a segregated manner. As such, individuals were happier if the information provided higher individual value (*vs*. the same information presented in the opposite manner).

Previous research has tested the hedonic framing hypothesis with results mainly consistent with theory (Thaler, 1985; Thaler and Johnson, 1990; Linville and Fischer, 1991; Cowley, 2008; Evers et al., 2016; Antonides and Ranyard, 2017). However, in the investment field, findings are mixed. For example, using sales records from 1991 to 1996 (i.e., non-experimental data), Lim (2006) evaluated investor preference for integrating or segregating outcomes to examine how capital gains and losses affect U.S. investors who sell multiple stocks on the same day. Result confirmed the hedonic framing hypothesis, namely, on the same day investors are more likely to bundle the sale of stocks trading below their purchase price (“losers”) with the sale of stocks trading above their purchase price (“winners”). In contrast, results from another study by Lehenkari (2009), using showed how real stock market data from the Finland Stock Market, did not support the main hypothesis. In that study only weak evidence for the hedonic framing hypothesis was found: investors in the sample did not consistently integrate losses and segregate gains, nor was there any significant preference for realized mixed gains rather than losses. These data indicate that the application of hedonic editing and prospect theory to the financial settings and investment field is not straightforward.

A second crucial factor in a mixed information condition, with both positive and negative information flows, is the order of the information. When evaluating the evidence and information in sequence, order and bias have been found to have an impact in various areas of accounting, auditing, and financial reporting (e.g., Ashton and Ashton, 1988, 1990; Pinsker, 2007, 2011; Daigle et al., 2015). Hogarth and Einhorn (1992) demonstrated a Belief-adjustment model and defined the order effect as follows: “There are two pieces of evidence, A and B. Some subjects express an opinion after seeing the information in the order A-B. Others receive the information in the order B-A. An order effect occurs when opinions after A-B differ from those after B-A” (see review by Kahle et al., 2005). In contrast, the Bayes' normative model, a Belief-adjustment model, suggests that more weight is placed on the most recent piece of evidence, with final judgements depending on the sequence of the provided evidence. This is especially true in situations characterized by an equal number of mixed information types (i.e., containing both positive and negative information). Psychological and behavioral studies have demonstrated that information order can affect individual decisions. More precisely, when requested to evaluate a short series with mixed information, the decision-maker will place more weight on the latest information. Thus, differential weighting of the information will result in a “recency effect” (Ashton and Ashton, 1988, 1990; Tuttle et al., 1997; Pinsker, 2007, 2011).

For investment decision-making, most investigations have used a stock price judgement task to assess the impact of a simple informational series. The series of information is processed either by sequential processing (i.e., SbS, Step by Step), where the evaluation will be done after receiving the information, or by simultaneous processing (i.e., EoS, End of Sequence), where the evaluation will be completed after receiving all of the information [Ashton and Ashton (1988, 1990), Pinsker (2004), see review by Kahle et al., 2005]. During the task, upon the subsequent release of each disclosure (if sequential) or set of disclosures (if simultaneous), subjects are asked to re-value the company's stock price (based on their prior valuation) and rate the direction of each disclosure on a scale from −10 (very bad news) to +10 (very good news). This approach confirmed the predictions of the Belief-adjustment. For example, Tuttle et al. (1997) examined the sequence effect on the efficiency of the market and concluded that individual investors experienced the recency effect when they received four clues or combined pieces of information. Pinsker (2004) examined stock price valuations from unsophisticated investors given different conditions of information. Continuous conditions (SbS) were significant in the direction of the information provided. While periodic (EoS) conditions were not significant at the midpoint of information evaluation, before all information had been provided. However, recency was detected after information items were released. Further evaluations were made for long series of clues and information. Pinsker (2011) conducted analysis of 40 information clues presented either simultaneously or sequentially: the results suggest a recency effect for all conditions, with significantly greater recency effect for the sequential conditions relative to the simultaneous conditions.

Two variables: Organization of Information (either Segregated or Integrated) and Sequence of Information (either negative-positive or positive-negative) have been shown to influence an individual's judgement and decision-making. However, these variables have not been assessed simultaneously. Nor have possible interaction and moderation effects been assessed for both the Organization of Information and the Sequence of Information during individual decision-making. Furthermore, the neural correlates of decision-making during these circumstances have not been investigated. More recently, according to Damasio's theories (Damasio, 1994, 1999), “emotions” have taken a principal role in the decision-making process and are a guide to the final executed choice. Supporting Damasio's studies, Bargh and Chartrand (1999) affirmed that, although humans are definitely capable of conscious deliberation, many economically relevant decisions rely on automatic, fast, and effective processes which are not under direct volitional control.

Herein, cerebral activity by electroencephalographic (EEG) recording, the galvanic skin response (GSR), and heart rate (HR) were evaluated during decision-making. In particular, Approach Withdraw (Davidson, 2004) and emotional (Mauss and Robinson, 2009; Vecchiato et al., 2014a) indices were used to estimate the “internal” state of the investigated subjects.

### Objectives

1. Test if the organization and order of information affect an individual's decision-making upon exposure to two pieces of information, simultaneously/separately and in a different order.
2. Evaluate the neuro-electrical correlates of decision-making during different experimental conditions. In particular, the neurometric correlates of the Organization of Information (two levels, either Segregated or Integrated) and Sequence of Information (two levels, either Negative-Positive or Positive-Negative) will be evaluated.

In this investigation, the behavioral level (individual's decision) and neurometric indices, via Emotional Index and Approach Withdraw Index (AWI), were used as dependent variables to measure the individual's decision when dealing with two pieces of mixed information (one piece of positive information and one piece of negative information) framed by different sequence and organization.

### Hypotheses

Three groups of mixed information, which are corresponding to hedonic editing hypothesis, were evaluated.

A. One piece of Small Positive Information and one piece of Small Negative Information. (SP/SN)

Hypothesis 1a: the Individual is willing to invest more/retire less money when receiving these two pieces of information integrated than when receiving the information in the Negative-Positive order.

Hypothesis 1b: the Individual has a more positive/less negative emotion when receiving these two pieces of information integrated than when receiving the information in the Negative-Positive order.

Hypothesis 1c: the Individual has a higher approach/lower withdraw tendency when receiving these two pieces of information integrated than when receiving the information in the Negative-Positive order.

B. One piece of Big Positive Information and one piece of Small Negative Information; (BP/SN)

Hypothesis 2a: the Individual is willing to invest more when receiving these two pieces of information integrated than when receiving the information in the Negative-Positive order.

Hypothesis 2b: the Individual has a stronger positive emotion when receiving these two pieces of information integrated than when receiving the information in the Negative-Positive order.

Hypothesis 2c: the Individual has higher approach tendency/lower withdraw tendency when receiving these two pieces of information integrated than when receiving the information in the Negative-Positive order.

C. One piece of Big Negative Information and one piece of Small Positive Information; (BN/SP)

Hypothesis 3a: the Individual is willing to retire less money when receiving these two pieces of information segregated than when receiving the information in the Negative-Positive order.

Hypothesis 3b: the Individual has a less negative emotion when receiving these two pieces of information segregated than when receiving the information in the Negative-Positive order.

Hypothesis 3c: the Individual has a lower withdraw potential when receiving these two pieces of information segregated than when receiving the information in the Negative-Positive order.

The information group: one piece of big positive information and one piece of big negative information is not tested in this research. It is mainly because this information group does not mentioned in original work of hedonic editing. Meanwhile, we consider this scenario relevantly rear in the actual world, especially in integrated situation: two important and dramatic events with the opposite valence happen at the same time point.

## Materials and methods

### Participants

Subjects were 20 Masters students from Sapienza Università di Roma, 23–26 years of age of which nine were females and 11 male. By self-report, all subjects indicated at least a basic knowledge of economics and finance and an understanding of basic terms: i.e., at least “3” out of “5” (0 for “none,” 3 for “basic,” 5 for “professional” levels of understanding). One subject was excluded in that he could not distinguish the size of information, i.e., big from small. Another subject's results were not complete due to technical issues. Hence, 18 valid results were analyzed (1 female and 1 male subject were excluded). Fourteen HR/GSR results were analyzed in that four HR/GSR results were missing.

Informed consent was obtained from each subject after explanation of the study, which was approved by the local institutional ethics committee. The experiment was conducted following the principles outlined in the Declaration of Helsinki of 1975, as revised in 2000 and was approved by the Sapienza University of Rome Ethical Committee in Charge for the Department of Molecular Medicine. All the subjects received extra credits in their course for participating this study. They were told that the performance of the experiments determined how much extra points they could receive.

### Stimulus material and procedure

Materials used were pieces of information that related to the state of a company. These materials were primarily derived from Tuttle et al. (1997), Pinsker (2007, 2011), and Daigle et al. (2015). All were properly translated into Italian. Each piece of information contained two to three sentences. Most information was presented in an auditing format including; operation loss/gain, negative cash flow/improving cash inflow from large customers, increasing/reducing costs, lost customer or market share/rapidly growing customer base, slow inventory turnover, product quality problem, expiration/gain of a patent on a key product, a likely infusion of equity capital, etc. The non-auditing format included layoff of employees, delay in releasing new products, and customer satisfaction.

Three groups of information were tested; a piece of Small Positive Information and a piece of Small Negative Information (SP/SN), a piece of Big Positive Information and a piece of Small Negative Information (BP/SN), and a piece of Big Negative information and a piece of Small Positive Information (BN/SP).

To determine how an individual's investment decision was affected by both Organization and Sequence of Information, a 2 × 2 within-subject design was used. The first examined variable was the Organization of Information, with a two-level manipulation. (1) Two pieces of Integrated information (0, two pieces of information). In these cases, the subject was first informed “no new information is released at the current moment by the company,” and then the subject was presented simultaneously with two pieces of information. (2) Two pieces of Segregated information (one piece of information and then the other piece of information). In these cases, the subject was presented with one piece of information at two separate time periods. The second manipulated variable was the Sequence of Information: Two pieces of information presented either in a Negative-Positive order (N-P) or in a Positive-Negative order (P-N). Table 1 summarizes all scenarios.

**Table 1:** Experiment design is listed for all three groups of information: SP/SN; BP/SN; BN/SP.

		**Organization of the information**	
		**Integrated**	**Segregated**
Sequence of information	Negative-Positive	(0, SN-SP) (0, SN-BP) (0, BN-SP)	(SN, SP) (SN, BP) (BN, SP)
	Positive-Negative	(0, SP-SN) (0, BP-SN) (0, SP-BN)	(SP, SN) (BP, SN) (SP, BN)

*All scenarios are listed*.

The adopted experimental task was similar to the Stock Price Judgement task used by Pinsker (2004, 2011). However, rather than asking the subjects to revalue the stock price, each subject was asked to make an investment decision, deciding how much of an investment would be made in the target company. With this behavioral task, a clearer indication of the individual's decision for each scenario was assessed.

At the beginning of each trial, subjects were informed that they had 1,000 Euro in cash and stock worth 1,000 Euro that have already been invested in Company A. Subjects were told that the Company will release a piece of information every 6 months. Subjects were then informed that two pieces of information related to the state of Company A would be provided. After reading the two pieces of information, the subjects had the opportunity to adjust their investment plan; (1) they could choose to invest more cash in Company A, (2) or they could choose to retire some or all of their money from the investment, (3) or they could make no change. The result of the decision, namely the total amount of money that was invested or retired was analyzed as the behavioral result (i.e., the main dependent variable measure).

The experiments were conducted on computer with software Presentation (Neurobehaviral Systems Inc., Presentation Research License). The experiments were carefully programed in Presentation according to following procedure. In all scenarios (both Segregated and Integrated situations for all three groups of information), subjects were given the first piece of information, with a maximum 20″ for reading. After reading, subjects pressed the key for the next page where they rated the information from −10 (very negative) to 10 (very positive) based on how positive or negative the information was perceived. Then subjects were then given the second piece of information to read and to rate from −10 (very negative) to 10 (very positive) based on how negative or positive the information was perceived. On the next page, subjects were asked to consider their reasons to either invest or retire, for 30 s. Afterward, subjects had 10″ to type their decision: i.e., how much money to invest or retire. In addition, they had 5″ to justify their decision in order to ensure that the protocol was followed (these were not used as dependent variables).

The presentation of each trial was randomized for each group of information (SP/SN, BP/SN, BN/SP). That is, for each of the three groups, the four Organization and Sequence possibilities were randomized (e.g., in the first group (SP, SN), the four possibilities were: 0, SN-SP; SN, SP; 0, SP-SN; SP, SN). EEG and HR/GSR measurements (see details below) were recorded throughout the experimental procedure.

### Manipulation check

The aim of this investigation was to test an individual's investment decisions based on information provided by differing organization and sequence patterns resulting from a series of statements either; very negative, small negative, very positive, or small positive. Initially it was necessary to measure how the subjects perceived the negativity or positivity of each received statement. For this reason, all statements had been previously validated through a manipulation check carried out with the same group of subjects. This procedure was conducted during a separate appointment 3–5 days before the experiment.

Subjects were required to read and to evaluate the materials by use of an international online platform, used in psychological research, that delivers questions and records answers via the Internet (Unipark©, made available from Sapienza Università di Roma's subscription at the Dipartimento di Psicologia dei Processi di Sviluppo e Socializzazione). After reading one piece of information, subjects rated the information from −10 (very negative) to 10 (very positive) based on how positive or negative the information was perceived (see Table 2 for details). Information rated between 8 and 10 was considered Big Positive Information; the information rated between 2 and 5 was considered Small Positive Information; the information rated between −10 and −8 was considered Big Negative Information; the information rated between −5 and −2 was considered Small Negative Information. Only these four types of information met the criteria for inclusion in the following experiment.

**Table 2:** Categorization and selection of information based on participant's rating score for each piece of information.

**Participant's rating range**	**Categorization of information**
Between 8 and 10;	Big Positive Information;
Between 2 and 5;	Small Positive Information;
Between −10 and −8;	Big Negative Information;
Between −5 and −2;	Small Negative Information;

### Neurometric data acquisition

#### EEG data

Subject's cerebral activity was recorded by means of a portable EEG system (BEPlus and Galileo software, EBneuro, Italy). Informed consent was obtained from each subject after explanation of the study, which was approved by the local institutional ethics committee. All subjects were comfortably seated on a reclining chair, in an electrically-shielded, dimly-lit room. Sixty four electrodes were arranged according to the 10–20 international system. Initially, recordings were extra-cerebrally referred and then converted to an average reference off-line. EEG activity was collected at a sampling rate of 256 Hz with the impedances below 5 kΩ. Each EEG trace was then converted into a Brain Vision format (BrainAmp, Brainproducts GmbH, Germany) for signal pre-processing and artifact detection, filtering, and segmentation. The EEG signals were band pass filtered at 1–45 Hz and depurated of ocular artifacts by Independent Component Analysis (ICA). The EEG data were re-referenced by computing the Common Average Reference (CAR). For each subject, the Individual Alpha Frequency (IAF) was estimated in order to define the frequency bands of interest according to the methods suggested by the relevant scientific literature (Klimesch, 1999).

#### Cardiac and galvanic skin response data

The considered autonomic activities, namely Galvanic Skin Response (GSR) and Heart Rate (HR), were recorded with the Nexus-10 system (Mind Media, Netherlands) with a sampling rate of 32 Hz. Skin conductance was recorded by the constant voltage method (0.5 V). Ag–AgCl electrodes (8 mm in diameter for active area) were attached to the palmar side of the middle phalanges of the second and third fingers of the participant's non-dominant hand by means of a velcro fastener. The company also provided disposable Ag–AgCl electrodes to acquire the HR signal. Before applying the sensors to the subject's skin, the surface was cleaned following procedures and suggestions previously published (Boucsein, 2012). GSR and HR signals were continuously acquired for the entire duration of the experiment and then filtered and segmented with in-house MATLAB software. For the GSR signal, a Continuous Decomposition Analysis was performed. The HR signal was obtained using the Pan Tompkins algorithm for the gathered electrocardiographic signals (Pan and Tompkins, 1985). In an attempt to match GSR and HR signals, the circumplex model for affect plane was used, where the coordinates of a point in space were defined by the HR (horizontal axis) that describes the valence phenomena and by the GSR (vertical axis) that describes the arousal phenomena (Russell and Barrett, 1999).

### Approach withdraw index (AWI)

Specific attention was given to the frontal scalp areas overlying the prefrontal cortices. Several studies (e.g., Davidson, 2004; Maglione et al., 2015; Cartocci et al., 2016a,b,c, 2017a,b,c,d; Cherubino et al., 2016a,b; Marsella et al., 2017; Modica et al., 2017) describe the frontal cortex as an area of interest for the analysis of this approach for withdrawal attitude (Davidson, 2004) and cerebral effort (Klimesch, 1999). In order to define AWI based on the EEG frontal asymmetry theory, imbalance was computed as the difference between the average EEG power of right and left recorded channels using the following formula.

(1) $$\begin{array}{l} AW=\;\frac{1}{N_{P}}\sum_{i\in P}x_{\alpha_{i}}^{2}\left(t\right)-\frac{1}{N_{Q}}\sum_{i\in Q}y_{\alpha_{i}}^{2}\left(t\right)=\\ \;=Average\;Power_{\alpha_{right,frontal}}-{Average\;Power}_{\alpha_{left,frontal}\;}. \end{array}$$

*x*_α_*i*__ and *y*_α_*i*__ represent the EEG channels in the alpha band that were recorded from the right and left frontal lobes, respectively. In addition, P and Q were two sets of EEG sensors employed and described as follows:

$$\begin{array}{l} \text{P}=\;\left\{\text{Fp}2,\text{ AF}6,\text{ AF}4,\text{ F}4\right\}\text{ and Q}=\;\left\{\text{Fp}1,\text{ AF}7,\text{ AF}3,\text{ F}5\right\}. \end{array}$$

*N*_*P*_ and *N*_*Q*_ represent the cardinality of these two sets of channels. According to Davidson's theory (Davidson, 2004), an increase in AWI will be related to an increase in the approach of the subject during decision-making and vice versa.

For each individual, the AWI signal was estimated during the entire decision-making time interval. Then, the signal was standardized by using the mean and variance of the AWI signal estimated during the baseline intervals recorded at the beginning and at the end of the experiment. The AWI of the entire experimental group was obtained by averaging the different standardized individual AWI.

Positive AWI values indicated an approach motivation toward the stimulus expressed by the subject, while negative AWI values, a withdrawal tendency.

### Emotional index

The emotional index was defined as previous described (Vecchiato et al., 2014b) by accounting for the GSR and HR signals for each individual during the decision-making task. For variables, an affect circumplex plane (Russell and Barrett, 1999) was constructed where emotions were defined in terms of arousal and their emotional valence. As a proxy for arousal, the values of the estimated GSR measurements and HR were used as a proxy for emotional valence. Standardized values for *GSR and HR* were then assessed by using the same baselines employed in the definition of the AWI index, using *GSRz* and *HRz* values for the generation of an Emotional Index *(EI)*. The *EI* was defined as follows:

(2) $$\begin{array}{l} EI=1-\frac{\beta }{\pi }. \end{array}$$

Where:

(3) $$\beta =\{\begin{array}{c} \frac{3}{2}\pi +\pi -\vartheta \;if\;GSR_{Z}\geq 0,\;HR_{Z}\leq 0\\ \frac{\pi }{2}-\vartheta \;otherwise \end{array}.$$

*GSR*_*Z*_ *and HR*_*Z*_ represent the z-score variables for GSR and HR respectively; ϑ, in radians, is measured as *arctang* (*HR*_*Z*_, *GSR*_*Z*_). Therefore, the angle β was defined in order to transform the domain of ϑ from [-π, π] to [0, 2 π] to obtain the EI varying between [−1, 1]. In this manner, β was calculated two ways. According to Equations 2 and 3 and the affect circumplex (Russell and Barrett, 1999), negative (*HR*_*Z*_ < 0) and positive (*HR*_*Z*_ > 0) values of the *EI* are related to negative and positive emotions, respectively, spanning the whole affect circumplex.

### Low resolution tomography (LORETA)

To evaluate the three-dimensional distribution of estimated cerebral activity, exact Low Resolution Tomography (eLORETA) software was used (Pascual-Marqui et al., 2011). eLORETA is a validated method for localizing the electric activity in the brain based on multichannel surface EEG recordings (Horacek et al., 2007; Pascual-Marqui et al., 2011, 2014; Müller et al., 2016). Such LORETA methodology was used to represent at the cortical level the results obtained for the whole EEG collected during the decision-making time period. No specific hypothesis was formulated for the estimated cortical activity by LORETA during decision-making by the subjects. However, results were used to describe significant effects. The cortical areas with spectral EEG power that differ significantly in particular frequency bands (Theta, Alpha) between two particular conditions are shown in color (from blue to red), while the cortical areas in which there was no statistical difference are shown in gray. Statistical analysis was performed taking into account the possibility for Type I errors, by using the Holm correction (Pascual-Marqui et al., 2011). The statistical tests were performed at a nominal significance of 5% (Holm corrected for multiple comparisons). A color code was used to depict statistical significance between A and B conditions: a red color is a significant increase in EEG power spectra, A vs. B, a blue color is not significant. It is worth noting that in the Alpha band, an increase in EEG power spectra was associated with a decrease in cortical activity in the frequency band, with the opposite true as well. In the Theta band, an increase in EEG power spectra was associated with an increase in cortical activity in the frequency band, with the opposite true as well (Pascual-Marqui et al., 2011).

### Statistical analysis and qualitative description

Statistical analyses were performed with SPSS 15.0 (SPSS Inc., 1989–2006, United States). Specifically, repeated-measures ANOVAs for factors (Organization of Information, Sequence of Information) were carried out for the behavioral measures (the total amount of investment) and for neurometric indices (i.e., AWI and EI). Greenhouse-Geisser corrections were applied if the assumption of sphericity was violated.

## Results

### One piece of small positive information and one piece of small negative information [SP/SN]

Table 3 reports the general results for the SP/SN information scenario. When subjects were given one piece of Small Positive Information and one piece of Small Negative Information, the variable integration/segregation did not show any statistical significance in the three indices: i.e., Investment, Emotional Index, and AWI. The variable order of information shows statistical significance for Investment and AWI, but no significance for Emotional Index, which suggests no emotional involvement during such a decision.

**Table 3:** Summary of statistical results for the information scenario SP/SN.

	**Investment**	**Emotion**	**AWI**
Integration/segregation	–	–	–
Negative-positive/positive-negative	X	–	X

“*X” indicates statistical significance for the measure.“-”indicates no statistical significance for the measure*.

**Figure 1:**
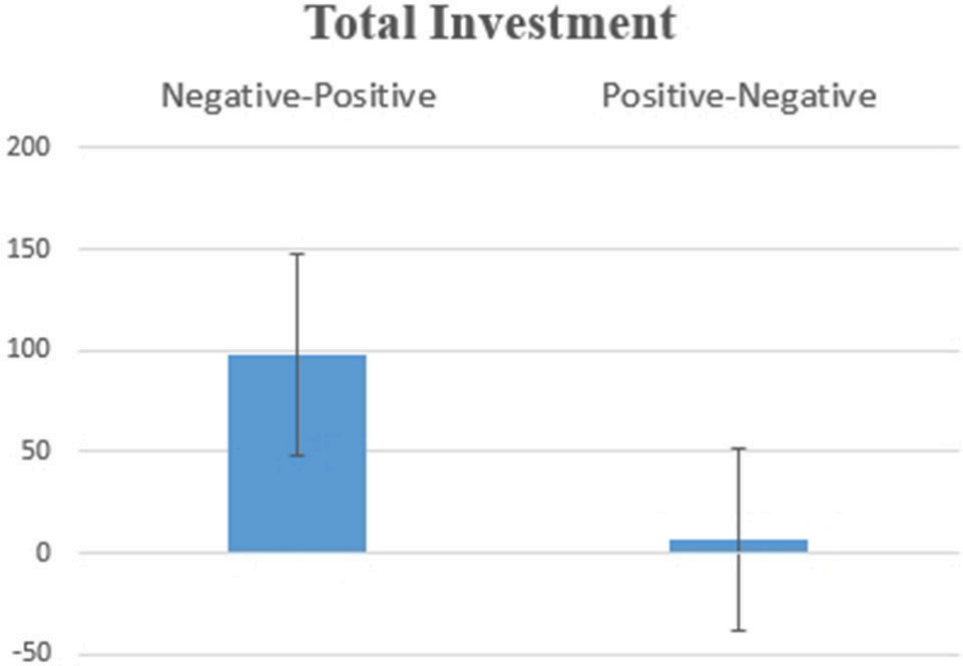
Average values for the total investment decision for SP/SN. The difference was statistically significant at *F*_(1, 17)_ = 4.711, *p* = 0.044.

Hypothesis 1a: Figure 1 shows the amount of total investment according to the Order of Information [*F*_(1, 17)_ = 4.711, *p* = 0.044]. This significant result indicates that the investment was higher when two pieces of information were received according to the order Negative-Positive (*vs*. Positive-Negative), suggesting a recency effect, which is consistent with the hypothesis.

Hypothesis 1b: For Emotional Index, there was no statistical significance due to either organization or the order of information.

**Figure 2:**
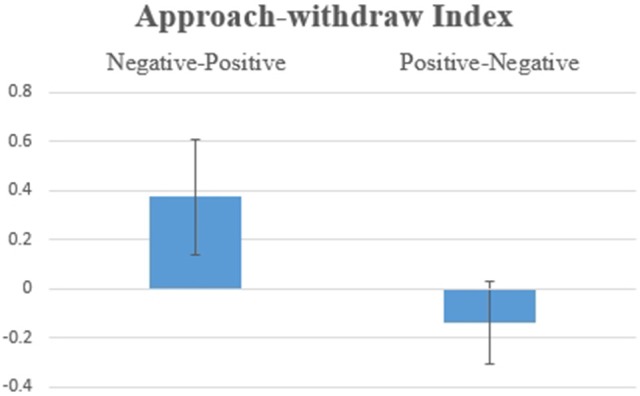
Average values for the Approach Withdraw Index for SP/SN: two pieces of information were presented in Negative-Positive vs. Positive-Negative order: differences were statistically significant at *F*_(1, 17)_ = 7.486, *p* = 0.014.

Hypothesis 1c: Figure 2 shows the Order of Information main effect [*F*_(1, 17)_ = 7.486, *p* = 0.014] for the Approach Withdraw Index: Subjects had higher average values for approach potentials when a piece of positive information was presented second, which confirms a recency effect.

**Figure 3:**
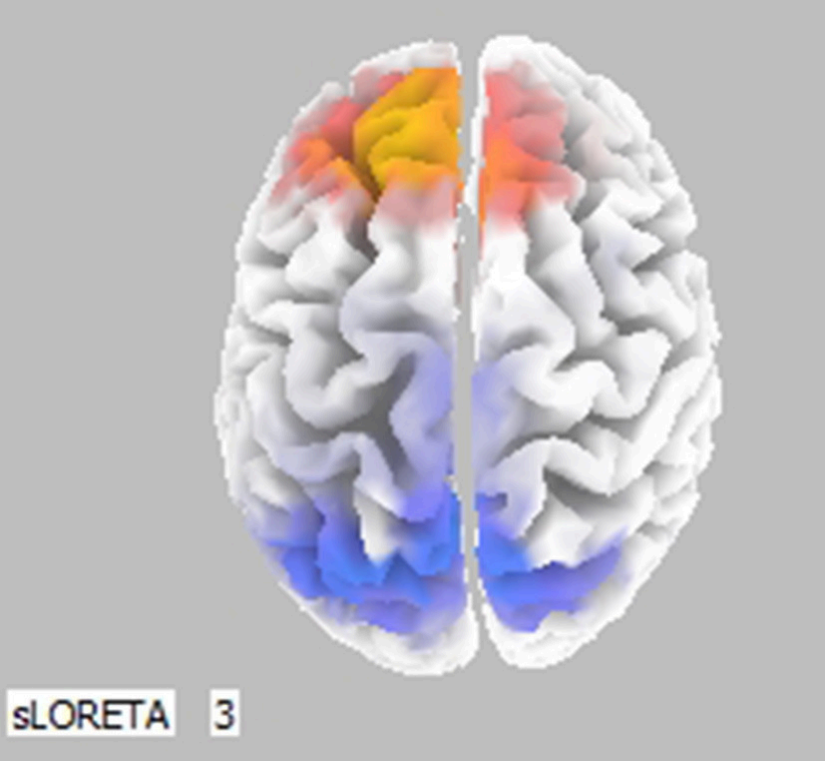
Statistically significant cerebral activity was estimated by LORETA software in the Lower Alpha Band comparing two pieces of information presented in scenario (0, SN+SP) and scenario (0, SP+SN). Orange areas suggest a significant increase in cortical activity for the first scenario (O, SN+SP) with respect to the other (0, SP+SN). Blue colors suggest the opposite.

Cortical activity was estimated by the LORETA approach (Figure 3, only frontal areas were assessed). Results show that comparison of (0, SN+SP) to (0, SP+SN) was coherent with Figure 2. When the information was integrated, there was lower activation in the left prefontal area compared to the right in the scenario (0, SN+SP) than in the (0, SP+SN) scenario. This result indicates a stronger withdrawal tendency in the presentation of (0, SP+SN) and comfirms the recency effect with subjects more influenced by the most recent information when the information was integrated. Specifically, when the second piece of information was positive, subjects had less withdrawal potential (orange colors in the frontal areas depicted in Figure 3).

**Figure 4:**
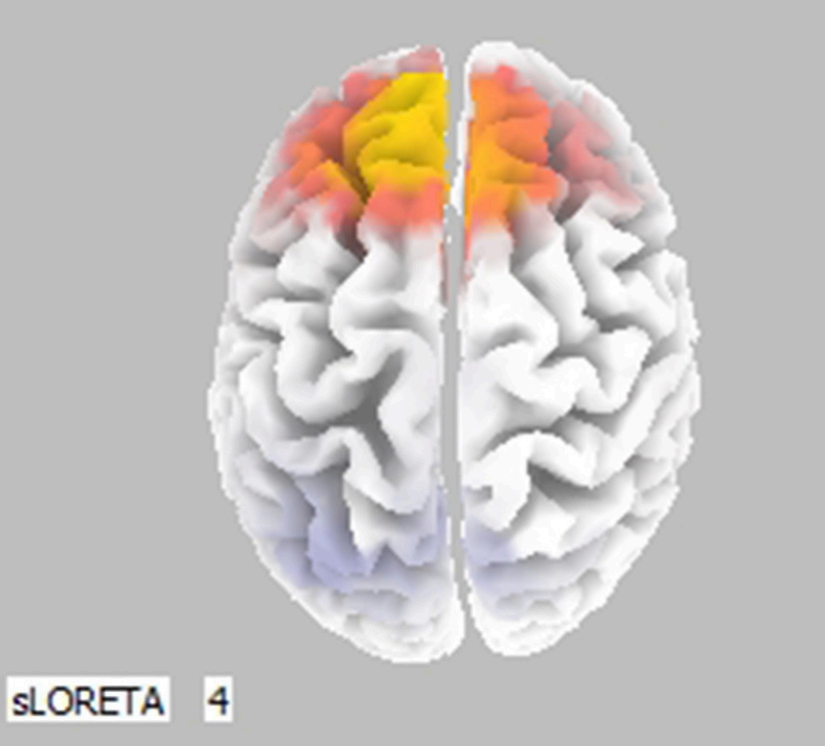
Statistically significant cerebral activity was estimated by LORETA software in the Upper Alpha Band comparing two pieces of information presented for scenario (0, SN+SP) with scenario (SN, SP). Orange areas suggest a significant increase in cortical activity for the scenario (0, SN+SP) with respect to the other scenario.

For the LORETA results (Figure 4), when (0, SN+SP) and (SN, SP) were compared in the Upper Alpha Band, there was lower activation in the left prefrontal area compared to the right in (0, SN+SP) than in (SN, SP). With (SN, SP) subjects have a stronger withdrawal tendency. This result is consistent with Thaler's prediction that with information types like SN/SP, it is better to provide integrated rather than segregated information, as segregation will result in higher withdraw potential.

### One piece of big positive information and one piece of small negative information [BP/SN]

Table 4 shows the general results for the information scenario BP/SN. When subjects were given one piece of Big Positive Information and one piece of Small Negative Information, the variable integration/segregation showed statistical significance in the biometric indices: Emotional Index and AWI, but no significance in the Behavioral Investment measure. The variable order of information showed statistical significance for the Investment measure but no significance for the biometric indices: Emotional Index and AWI.

**Table 4:** Summary of statistical results for the information scenario BP/SN.

	**Investment**	**Emotion**	**AWI**
Integration/segregation	–	X	X
Negative-positive/positive-negative	X	–	–

“*X” indicates statistical significance for the measure.“-”indicates no statistical significance for the measure*.

**Figure 5:**
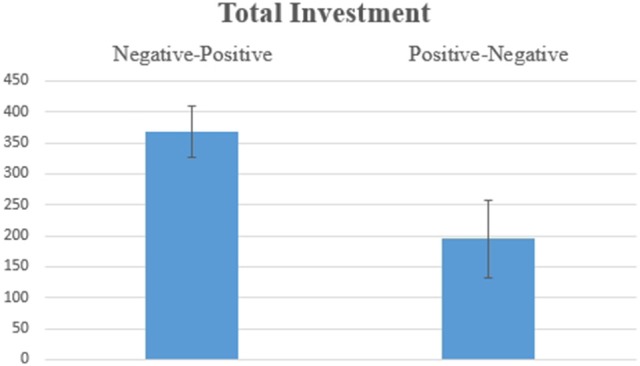
Average values for the total investment decision for BP/SN. The difference was marginally significant at *F*_(1, 17)_ = 3.615, *p* = 0.074.

Hypothesis 2a: Figure 5 shows, for the amount of total investment, a marginally significant [*F*_(1, 17)_ = 3.615, *p* = 0.074] effect for the Order of Information. This result indicates that investment tends to be higher when the two pieces of information are in the order Negative-Positive, suggesting a tendency for a recency effect, which is partially consistent with the hypothesis.

**Figure 6:**
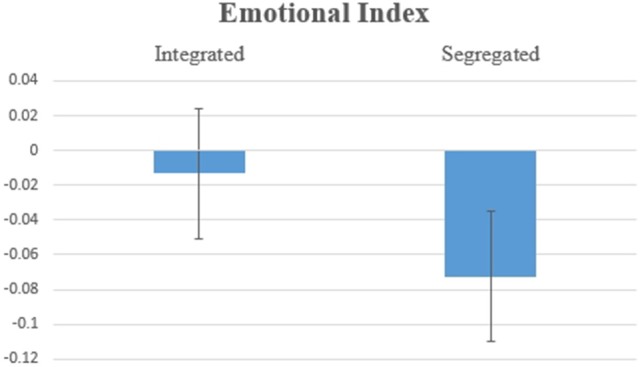
Average values for Emotion Index for BP/SN, two pieces of information integrated vs. segregated. The difference was statistically significant at *F*_(1, 13)_ = 8.574, *p* = 0.012.

Hypothesis 2b: Figure 6 shows the results for Emotional Index, with a significant effect for the variable Organization of Information [*F*_(1, 13)_ = 8.574, *p* = 0.012]. This result suggests higher Emotional Index when one piece of Big Positive Information and one piece of Small Negative Information are integrated.

**Figure 7:**
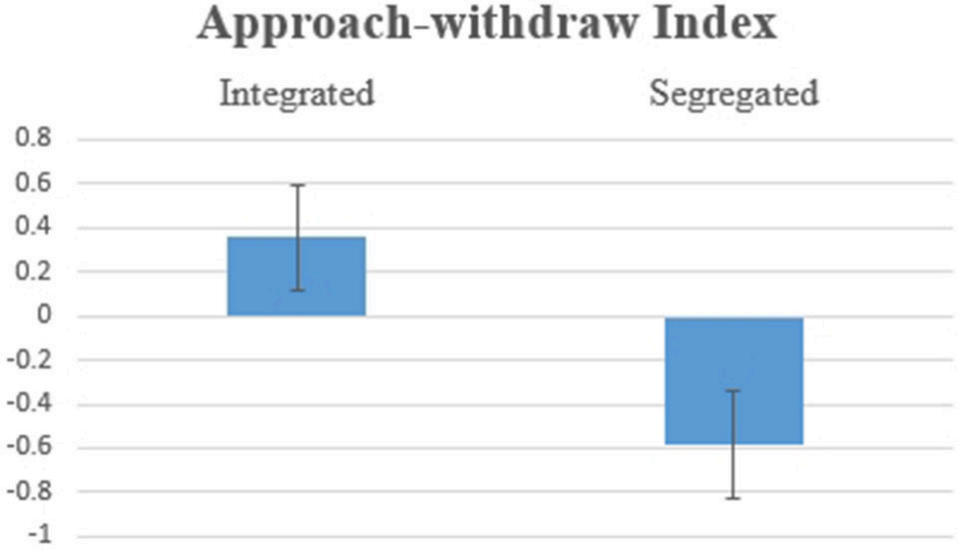
Average values for the Approach Withdraw Index for BP/SN, two pieces of information integrated vs. segregated. The difference was statistically significant at *F*_(1, 17)_ = 11.373, *p* = 0.004.

Hypothesis 2c: The AWI was consistent with the Emotional Index, showing a significant effect [Figure 7; *F*_(1, 17)_ = 11.373, *p* = 0.004] for the variable Organization of Information. In this information group, the average for the Integrated Information condition was higher than the average for the Segregated Information condition.

**Figure 8:**
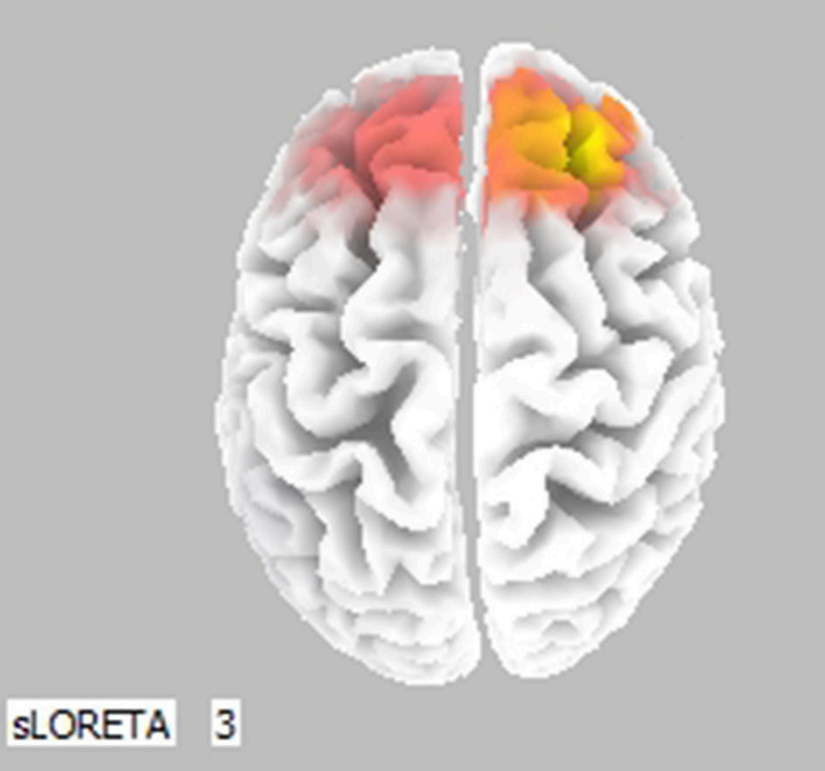
Statistically significant cerebral activity was estimated by LORETA software for the Lower Alpha Band for comparison of two pieces of information in (SN, BP) and in (0, SN+BP). A similar result was generated in the Upper Alpha Band. Orange areas suggest a significant increase in cortical activity for the first scenario (SN, BP). For this experimental condition, there were no important difference in the cortical activities estimated by the two different frequency bands.

For the alpha band in the LORETA map (Figure 8), the lower activation in the right prefrontal area was observed by compariosn of (SN, BP) to (0, SN+BP). The result suggests a higher approach tendency when information was integrated in the Negative-Positive order.

**Figure 9:**
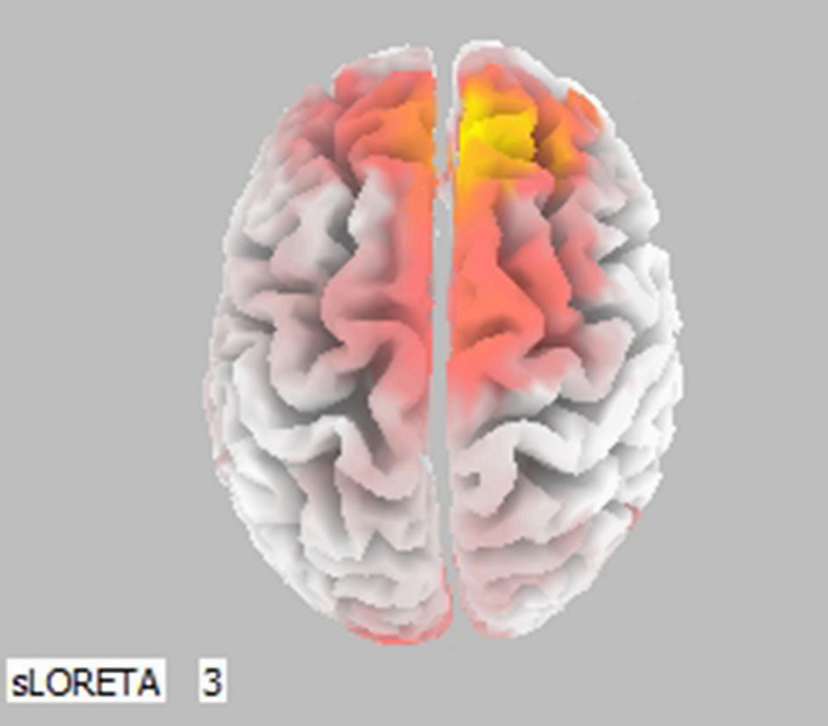
Statistical significant cerebral activity was estimated by LORETA software in the Lower Alpha Band comparing two pieces of information presented in (0,BP+SN) with (0, SN+BP). A similar result was generated in the Upper Alpha Band. Orange areas suggests a significant increase in cortical activity for the first scenario (0, BP+SN).

Another significant LORETA map effect (see below, Figure 9) was found by comparison of (0, BP+SN) to (0, SN+BP). A lower activation in the right prefontal area was observed when comparing the scenario (0, BP+SN) to (0, SN+BP). Results indicated a recency effect when information was integrated. A stronger approach tenedncy was observed when information was presented Negative-Positive rather than Positive-Negative.

### One piece of big negative information and one piece of positive information [BN/SP]

Table 5 shows results for BN/SP. When subjects were provided one piece of BN information and one piece of SP information, variable integration/segregation showed a statistically significant effect for the Biometric Indices, especially for the Emotional Index. No significant effect for the Investment Behavioral measure was observed. The variable Order of Information showed a statistically significant effect for the Investment measure but no significant effect for the Emotional Index. For the Approach Withdraw Index, there was an interaction between the two variables.

**Table 5:** Summary of statistical results for BN/SP.

	**Investment**	**Emotion**	**AWI**
Integration/segregation	–	X	Interaction
Negative-positive/positive-negative	X	–	

“*X” indicates a statistically significance effect for the variable.“-”indicates that no statistical significance was shown for the variable*.

**Figure 10:**
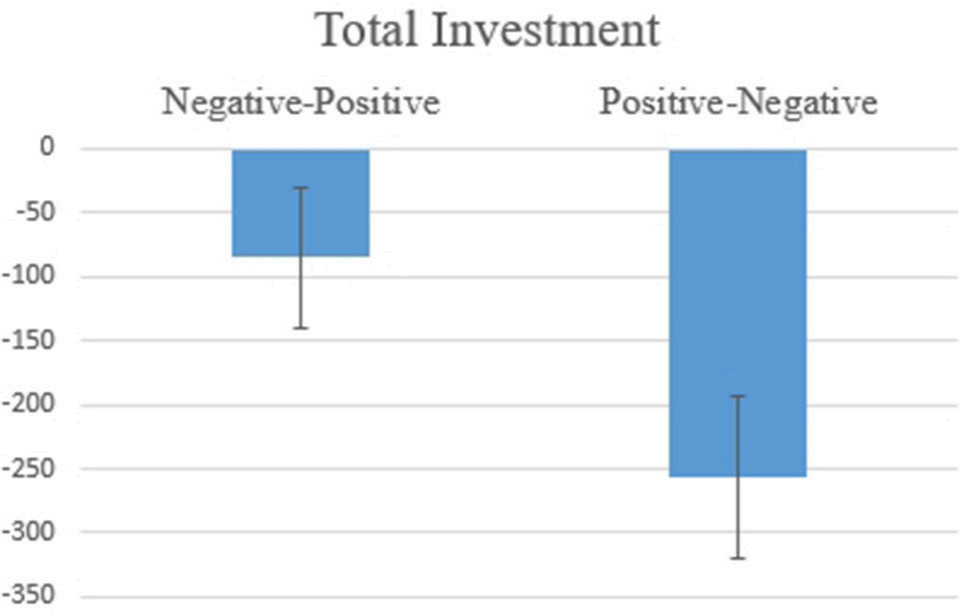
Average values for the total Investment decision for BN/SP. The difference was statistically significant at *F*_(1, 17)_ = 4.590, *p* = 0.047.

Hypothesis 3a: The amount of total investment showed a significant effect [*F*_(1, 17)_ = 4.590, *p* = 0.047, Figure 10] for the Order of Information variable. The results indicate that investment was higher when two pieces of information were in the order Negative-Positive, suggesting a recency effect, which is partially consistent with the hypothesis.

**Figure 11:**
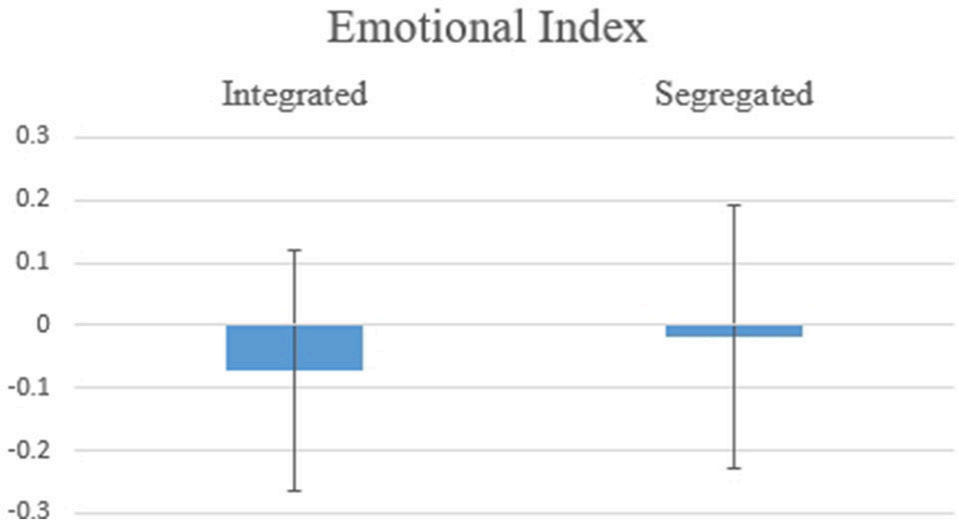
Average values for the Emotional Index for BN/SP, two pieces of information Integrated vs. Segregated. The difference was statistically significant at *F*_(1, 13)_ = 4.871, *p* = 0.046.

Hypothesis 3b: Fort the Emotional Index, there was a significant effect for the variable Organization of Information [*F*_(1, 13)_ = 4.871, *p* = 0.046, Figure 11], which is consistent with Thaler's principle: on average, individuals feel a less negative emotion when receiving two pieces of information Segregated instead of Integrated (herein a big negative and a small positive).

**Figure 12:**
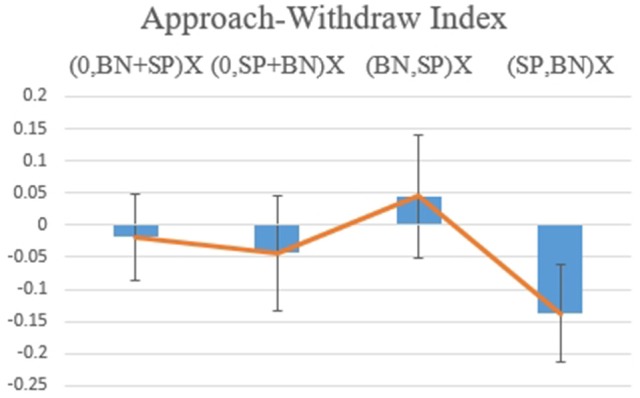
Value for the Approach Withdraw Index for (0, BP+SN), (0, SN+BP), (BP, SN), and (SN, BP).

Hypothesis 3c: For the AWI, there was marginal significance for the interaction between Organization of Information and the Order of Information [*F*_(1, 17)_ = 3.720, *p* = 0.071, Figure 12]. When the information was provided in the order Negative-Positive, the individual had a stronger willingness to approach when two pieces of information were presented separately rather than simultaneously [*F*_(1, 17)_ = 5.473, *p* = 0.032]. Marginal significance [*F*_(1, 17)_ = 3.892. *p* = 0.065] for the recency effect was observed when two pieces of information were segregated: Negative-Positive order was preferred when compared to Positive-Negative.

**Figure 13:**
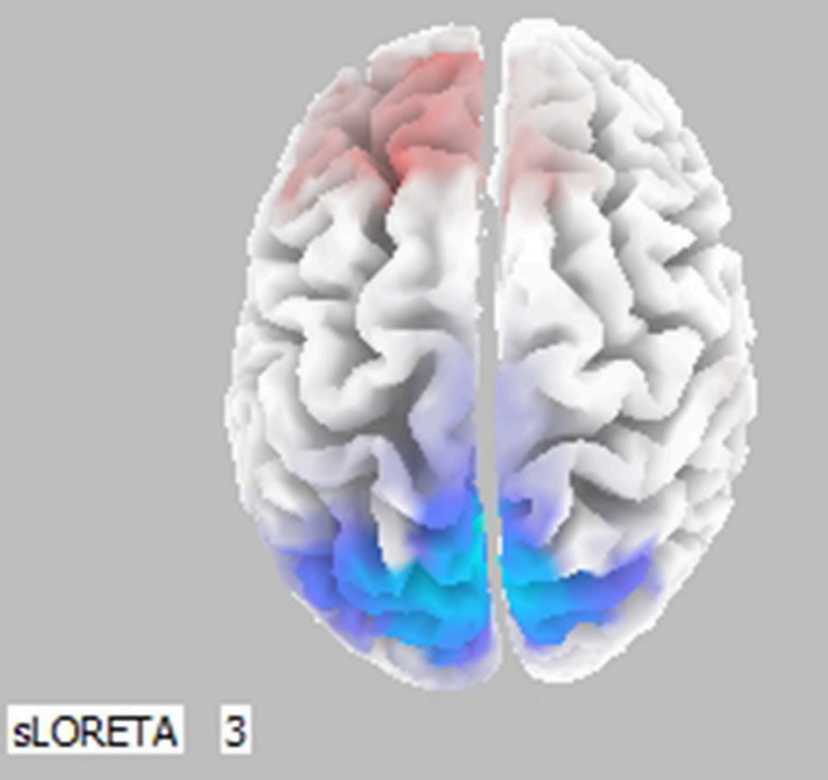
Statistically significant cerebral activity was estimated by LORETA software in the Lower Alpha Band when two pieces of information were presented in (SP, BN) and in (0, SP+BN). Similar results were observed in the Upper Alpha Band. Orange areas suggest a significant increase in cortical activity for the first scenario (SP, BN) with respect to the other scenarios. Blue colors depict the opposite.

For the LORETA map (Figure 13), there was significant Alpha Band activation in the prefrontal area for comparisons of (SP, BN) with (0, SP+BN). (0, SP+BN) had a higher withdraw potential when compared to (SP, BN). The result suggests that with a Positive-Negative order, it is better separate the information rather than integrate it, which is consistent with Thaler's theory.

## Discussion

Humans are influenced by unrecognized and finely tuned affective mechanisms, which often play a decisive role in decision-making and action (Davidson and Irwin, 1999; Panksepp, 2004). Many of these processes were shaped by evolution to serve social purposes (Cacioppo and Berntson, 2002; Adolphs, 2008; Astolfi et al., 2011) while decision-making and evaluation in economic contexts are influenced by mechanisms dedicated to social interaction. In this study, we investigated the neuro-electric correlates of decision-making during differing experimental conditions related to the temporal presentation of economic information.

Based on Thaler's hedonic editing framework and the Belief-adjustment model, the influence of the Organization of Information (Integration vs. Segregation) and the Sequence of Information (Negative-Positive order vs. Positive-Negative order) on decision-making both at the behavioral level (decision) and neurometrix level (measured by an individual's emotion and Approach Withdraw tendency) was assessed for the three groups of information: SP/SN, BP/SN, BN/SP. The results of this study partially verified the hypotheses.

The behavioral results, which are an individual's final investment decision, were consistent for all three scenarios (SP/SN, BP. SN, BN/SP, see Figures 1, 5, 10 in Results). In general, individuals will invest more/retire less when receiving two pieces of information in a Negative-Positive order. This result confirms previous findings (Ashton and Ashton, 1988, 1990; Tuttle et al., 1997), which suggest that for a short-series of mixed information, the subject's judgement exhibits the recency effect. For SP/SN, individuals invested 9.2% more when receiving two pieces of information in a Negative-Positive order than in a Positive-Negative order (average investment of 98.3 Euro vs. an average investment of 6.8 Euro). For BP/SN, individuals invested 36.8% more when receiving two pieces of information in a Negative-Positive order than in a Positive-Negative order (average investment of 367.9 Euro vs. average investment of 195.0 Euro). The amount of investment in a Negative-Positive order was 47.0% higher than in a Positive-Negative order. For BN/SP, individuals retired 17.2% less stock when receiving two pieces of information in a Negative-Positive order than in Positive-Negative order (average retirement of 84.8 Euro vs. average retirement of 257.1 Euro). The amount of retirement in Negative-Positive order was 67.0% lower than in a Negative-Positive order. From the behavioral results, the influence of information sequence was clear. The recency effect was found in all three groups. However, for the behavioral results, no significant difference was observed between the scenarios which two pieces of information were received Segregated or Integrated.

However, the neurometric results (Emotional Index, Approach Withdraw Index and results from LORETA) show differences among information groups. An effect of the Sequence of Information and the Organization of Information was found for the different scenarios.

For SP/SN, which represents an ordinary situation without large scale information, results show no statistical significance for Emotional Index. This result indicates less emotional involvement when information is small, suggesting a cognitive level of coping during such a scenario. Approach Withdraw Index, shows consistency with the behavioral results. The Order of Information, in particular the most recent information, affects an individual's decision: positive information immediately before the decision results in higher investment and a stronger approach potential. Whereas negative information immediately before the decision results in a stronger withdraw tendency and a lower investment/retirement of the money. This result is consistent with previous investigations that demonstrated the importance of information recency (Pinsker, 2004). The LORETA map comparing (0, SN+SP) to (0, SP+SN) confirms that when the second piece of information was negative, subjects had a stronger withdraw tendency. These results further stress the importance of the recency effect for not only behavioral decision making measures, confirming previous literature (Ashton and Ashton, 1988, 1990; Tuttle et al., 1997) but also for automatic cognitive processing measures, which is an innovative contribution of this investigation.

Moreover, these results suggest that for small pieces of negative and positive information, the order of information overrules the effect of the organization. Segregation or Integration of two small pieces of information does not affect an individual's investment decision as the hedonic editing theory predicts. With one exception for the LORETA map when comparing (0, SN+SP) to (SN, SP), the results indicate the best strategy Integration rather than Segregation in a Negative-Positive order, consistent with Thaler (1999) theory. Overall, the order of small scale information received determines priority in decision-making.

In the second and third hypotheses, the focus was on large-scale information. There are two asymmetrical cases; (1) one piece of Big Positive Information and one piece of Small Negative Information, (2) one piece of Big Negative Information and one piece of Small Positive Information. For these cases, Emotional Index influenced decision-making at the level of the individual's Approach Withdraw potential.

For BP/SN, the Emotional Index was significant as a variable of the Organization of Information confirming Thaler's theory concerning the integration/segregation of such pieces of information where positive emotion is higher in an Integrated vs. a Segregated condition. AWI results were consistent with Emotional Index results, indicating a higher approach potential for Integrated conditions. Thus, with a piece of Big Positive Information and a piece of Small Negative Information, it is best to integrate the two in order to boost both emotion and approach potentials. Support comes from the LORETA map, when (SN, BP) was compared to (0, SN+BP). When information was presented in the Negative-Positive order, Integration had a higher approach tendency. Nevertheless, even though AWI showed no significance for the effect of order, the LORETA map comparing (0, BP+SN) to (0, SN+BP) suggests the influence of order in this case. When information was integrated, Negative-Positive Order was favored over Positive-Negative. When big positive information was received after small negative information, subjects had a higher approach tendency, consistent with a recency effect.

For all four scenarios of BN/SP, emotion was negative, which may be due to stronger negative information rather than positive, with the perception that overall information was negative. Emotional Index was influenced by the Organization of Information with negative emotion higher when integrated. In other words, to diminish the emotional impact of Big Negative Information, it should be Segregated. AWI shows a similar trend, although significance depends on interaction between order and organization, indicating a preference for approach tendency. Segregation plus order are influenced by the recency effect, with Small Positive Information best when presented last. By LORETA map of the whole brain, a frontal alpha asymmetry was demonstrated with a Positive-Negative order. The results suggest that with a Positive-Negative order there is higher withdraw tendency when information is presented segregated than Integrated. This result is consistent with Thaler's prediction that a small reduction in loss should be presented in a Segregated manner.

For big scale information, emotions are aroused, influencing decision-making. Thaler's theory was confirmed by the Emotional Index results, indicating that a piece of big positive information and a piece of Small Negative Information result in more positive emotions, while segregating a piece of Big Negative Information and a piece of Small Positive Information results in less negative emotions. These Emotional Index results support hedonic editing from a biometric perspective. Based on Thaler's theory, individuals arrange multiple events, in both financial and non-financial/social domains, either separately or together, in order to maximize positive emotions. (e.g., Linville and Fischer, 1991; Hsee and Leclerc, 1998; Hsee and Zhang, 2004). This investigation demonstrates that a similar effect is observed for Emotional Index as well, with Segregation and Integration showing different valence and arousal for the individual's emotions.

Results from the Approach Withdraw Index and LORETA map show that both the Organization of Information, either Segregation or Integration, and the Order of Information, either Negative-Positive or Positive-Negative, affect a subject's evaluation. With a piece of Big Positive Information and a piece of Small Negative Information, Integration of the two pieces is preferred, which is consistent with Thaler's theory. Further, a recency effect is found when information is integrated, which is consistent with the Belief-adjustment model (Hogarth and Einhorn, 1992) and the recency effect (Tuttle et al., 1997; Pinsker, 2004; Ashton and Ashton, 1988, 1990). These results suggest that subjects reach the highest level of evaluation not only by presentation of an integrated cancellation in gain but also in a Negative-Positive order with the latest positive information providing a higher approach tendency. Similar results were obtained for one piece of Big Negative Information and one piece of Small Positive Information. The interaction of organization and recency suggest a small reduction in loss is preferred to a negative-positive order.

There are limitations to this investigation. First, the sample size is small, especially for the behavioral results and as well future investigations should consider individual variables. Hedonic editing theory often does not consider individual variables, yet the Belief-adjustment model has demonstrated individual psychological factors (e.g., initial beliefs, years of experience) are associated with belief revision effects in accounting (see review by Kahle et al., 2005). Future research should evaluate professional investors and to determine if professionals experience effects similar to non-professionals. Second, further study should consider using real money and using the respondents' own money during the experiments. Real money verse hypothetical payment treatment have been implemented in various laboratorial setting. The phenomenon, known as house money effect, has been observed in some cases. Participants are more risk-seeking and will spend more money when provided with a certain amount of money during the experiment (Cherry et al., 2005; Frino et al., 2008; Chang et al., 2009; Hensher, 2010 p.737). Likewise, decision-making should be evaluated in a variety of areas (e.g., consumer behavior) to identify similar effects and influences.

## Conclusion

In our paradigm, we considered and tested both the Organization of Information and the Order of Information using both behavioral and neurometric indices. The results are consistent with the Belief-adjustment model in which an individual's investment decision is influenced most by recent information when received in a short-series of mixed information. The neurometric results provide insight into the emotion and tendencies during the judgement procedure. The results suggest that in the scenarios that involve large-scale information, the organization of information (Integration vs. Segregation) influences the emotion and Approach Withdraw tendency, partially consistent with Thaler's hedonic editing theory. Where a big piece of information is involved in the scenario (either BP/SN or BN/SP), emotion is affected by organization in a manner consistent with the predictions of Thaler (1985), such that decreased gain should be presented as integrated, while a small reduction in loss should be presented as segregated. Presenting information in these ways provides for a higher positive emotional value. Moreover, for BP/SN, there is a greater approach potential when two pieces of information are integrated; while in the case of BN/SP, individuals favor the separation of information with the order Negative-Positive. A piece of Big Negative Information followed by a piece of Small Positive Information is the best way to create approach potentials.

The results of this investigation should provide insight for effective communication of information, especially when large-scale information is involved. For the communication of large scale information to individuals or investors, corporations should consider both the order and the organization of the information. Even though the final investment decision may not be different, the individual's emotion and tendency during judgement may be. For a small piece of positive information and a small piece of negative information, it is better to present them in the Negative-Positive order. For a piece of Big Positive Information and a small piece of negative information, it is better to present them in Negative-Positive order and present them Integrated. For a piece of Big Negative Information and a small piece of positive information, it is better to present the Big Negative Information first and the Small Positive Information second.

## Author contributions

WY has done majority part of the work, including doing literature review, experiment design, collecting data, analysing the data, interpretation of the result and writing the article. JM came up the main idea of this research and participated in the experiment design and the interpretation of the result. HC assisted in the experiment design and data collection. AM, EM, and DR assisted in data collection and data transfer prior to the analysis. GC assisted in the analyzing the data. MB assisted in preparing the material (translating English materials into Italian and back-translation), interpretation of the the result and provided critical revision. FB assisted in preparing the material (translating English materials into Italian and back-translation) and provided critical revision.

### Conflict of interest statement

The authors declare that the research was conducted in the absence of any commercial or financial relationships that could be construed as a potential conflict of interest.
